# Effects of managing fecal consistency on body weight in rats given liquid diets with pectin via tube feeding

**DOI:** 10.1186/s13104-025-07363-4

**Published:** 2025-07-16

**Authors:** Sachi Fukunaga, Erika Mori, Sho Miyatake, Ippei Yamaoka

**Affiliations:** Medical Foods Research Institute, Otsuka Pharmaceutical Factory, Inc, OS-1 Division, Tokushima, 772-8601 Japan

**Keywords:** Enteral nutrition, Body mass, Pectin, Diarrhea, Fecal condition

## Abstract

**Objectives:**

Tube feeding is a method of nutritional management that enables the maintenance of gastrointestinal function, even in patients who have difficulty with oral nutritional intake. Diarrhea is a major digestive symptom in patients receiving tube feeding, and may affect the maintenance of body weight. Therefore, this study aimed to investigate the effects of the deterioration of fecal consistency during tube feeding on body weight. We prepared 10 types of concentrated liquid diets with different contents of pectin, a dietary fiber that has been reported to maintain normal fecal consistency, and each diet was assigned to a separate group of rats with no overlap between the groups. We tube-fed each diet to gastrostomized rats for 5 days to investigate the effects of fecal consistency on body weight and nutritional status.

**Results:**

Rats fed a pectin-containing liquid diet had lower fecal consistency scores and a greater amount and rate of body weight gain than those fed a liquid diet without pectin. Furthermore, fecal consistency showed a significant correlation with the amount and rate of weight gain. These findings suggest that the maintenance of close to normal fecal consistency under tube-feeding management contributes to body weight gain during the feeding period.

**Supplementary Information:**

The online version contains supplementary material available at 10.1186/s13104-025-07363-4.

## Introduction

While tube feeding is advantageous in maintaining gastrointestinal function even in patients requiring parenteral nutrition, the risk of digestive issues remains a concern [1]. Diarrhea, a major gastrointestinal symptom, has a high incidence of 12–68% in patients receiving tube feeding [2], and it likely causes nutrient malabsorption [3, 4]. Because fecal consistency is associated with the quality of life (QoL) of patients, many studies have been conducted to improve fecal consistency in patients receiving enteral nutrition who have gastrointestinal symptoms. Many have only reported changes in fecal consistency during tube feeding, and few studies have investigated the changes in body weight before and after the improvement of digestive symptoms. Patients with inflammatory bowel disease may experience body weight loss along with diarrhea [5]. Therefore, we hypothesized that the deterioration of fecal consistency during tube feeding affects body weight and nutritional status.

The aim of this study was to investigate the effects of the deterioration of fecal consistency during tube feeding on body weight gain in rats. The liquid diets used in this study contained different amounts of pectin. Pectin, which is a water-soluble dietary fiber extracted from citrus fruits such as lemons and limes, has gel-forming ability under low pH conditions by forming cross-linked structures via divalent metal ions [6, 7]. Because pectin-containing enteral nutrition agents have been reported to maintain normal fecal consistency in rats [8], we assumed that the use of pectin-containing liquid diets was appropriate for improving fecal consistency in this study.

## Main text

### Materials and methods

#### Experimental animals and study design

This study adheres to the 4Rs (Reduce, Refine, Replace, and Responsibility) principles. Seven-week-old male Crl: CD (Sprague Dawley) rats (Jackson Laboratory Japan Inc., Japan) were housed at 23 ± 3 °C and 55 ± 15% humidity under a 12-h light/dark cycle (7:00 to 19:00). They were acclimated for at least 7 days before being included in the study, divided into 10 groups based on body weight, and fasted for 18 h. Gastrostomy was performed to create a gastric tube feeding route under isoflurane (Merck & Co., Inc., USA) anesthesia. An incision of approximately 3 cm was made along the midline of the abdomen, slightly below the sternum, to expose the stomach. A hole was made in the stomach fundus, through which the tip of a 6.5 fr Kangaroo™ enteral feeding tube (Cardinal Health, USA) was inserted. The tube was secured by suturing around the insertion site of the stomach with a 6 − 0 suture. The other end of the tube protruded through the abdominal cavity and passed subcutaneously on the back. An incision of approximately 5 mm was made between the scapulae, through which the tube exited the body. The abdomen was sutured through the muscle layer with a 3 − 0 suture, and the skin was closed with staples.

A commercially available liquid diet (HINEX E-Gel, Otsuka Pharmaceutical Factory, Inc., Japan) (Table S1) was supplemented with pectin in increments of 0.1 g/100 kcal to produce a total of 10 liquid test diets ranging from 0 to 0.9 g/100 kcal pectin. These were provided via gastric tube feeding to the 10 groups of rats (P0–P0.9 groups, *n* = 7–9). The P0 group received liquid diet without pectin. The day of gastrostomy surgery was defined as day 1 of feeding (Day 1), and the feeding of each liquid diet was started at 4:00 p.m. Considering the reduction in gastrointestinal function after fasting and surgery, the rats on Day 1 were given a dose of 40 kcal/day, which was delivered continuously for approximately 9 h at a rate of 4.5 mL/h. On Day 2 and subsequent days, a dose of 80 kcal/day, equivalent to the amount of calories consumed per day under ad libitum feeding conditions, was given continuously for 16 h at a rate of 5.0 mL/h. On Day 5, the body weight of all animals was measured. Subsequently, under 2% isoflurane, in air blood samples were collected from the abdominal aorta, and the rats were killed by exsanguination through severance of the abdominal aorta.

#### Fecal scoring and blood analysis

Fecal consistency was evaluated daily during the feeding period, with a score of 0 points given for normal feces, 1 point given for soft feces, 2 points given for muddy feces, and 3 points given for watery feces [9]. Scores were calculated each day by dividing the total score by the number of times feces were passed every day.

In this study, nutritional status was evaluated by blood chemistry tests. The blood samples were aliquoted into blood collection tubes containing lithium heparin and quickly cooled on ice. These samples were then centrifuged (4 °C, 3,000 rpm, 10 min) to separate the plasma, which was used for blood biochemical testing (the tests were outsourced to Nagahama Life Science Laboratory, Oriental Yeast Co., Ltd., Japan). Blood analysis was performed to evaluate the nutritional status. The parameters measured were albumin (ALB), blood urea nitrogen (BUN), chlorine (Cl), creatinine (CRE), inorganic phosphorus (IP), potassium (K), sodium (Na), and total protein (TP).

#### Statistical analysis

The results are expressed as mean ± standard deviation. A one-way ANOVA was performed to examine the effects of pectin contents in liquid diets, and a post-hoc test was performed for items that showed significant differences. For comparisons with the P0 group (control group), equal variance analysis was performed using Bartlett’s test. For the analysis, Williams’ multiple comparison test (two-sided test) was used if the variances were equal, and the Shirley–Williams multiple comparison test (two-sided test) if the variances were unequal. The correlation between body weight and fecal scores was analyzed using Spearman’s correlation coefficient. Statistically significant differences were considered at *p* < 0.025 in Williams’ multiple comparison test or 0.05 otherwise. Statistical processing was performed using SAS 9.4 (Takumi Information Technology Inc., Japan).

## Results

**Fig. 1:**
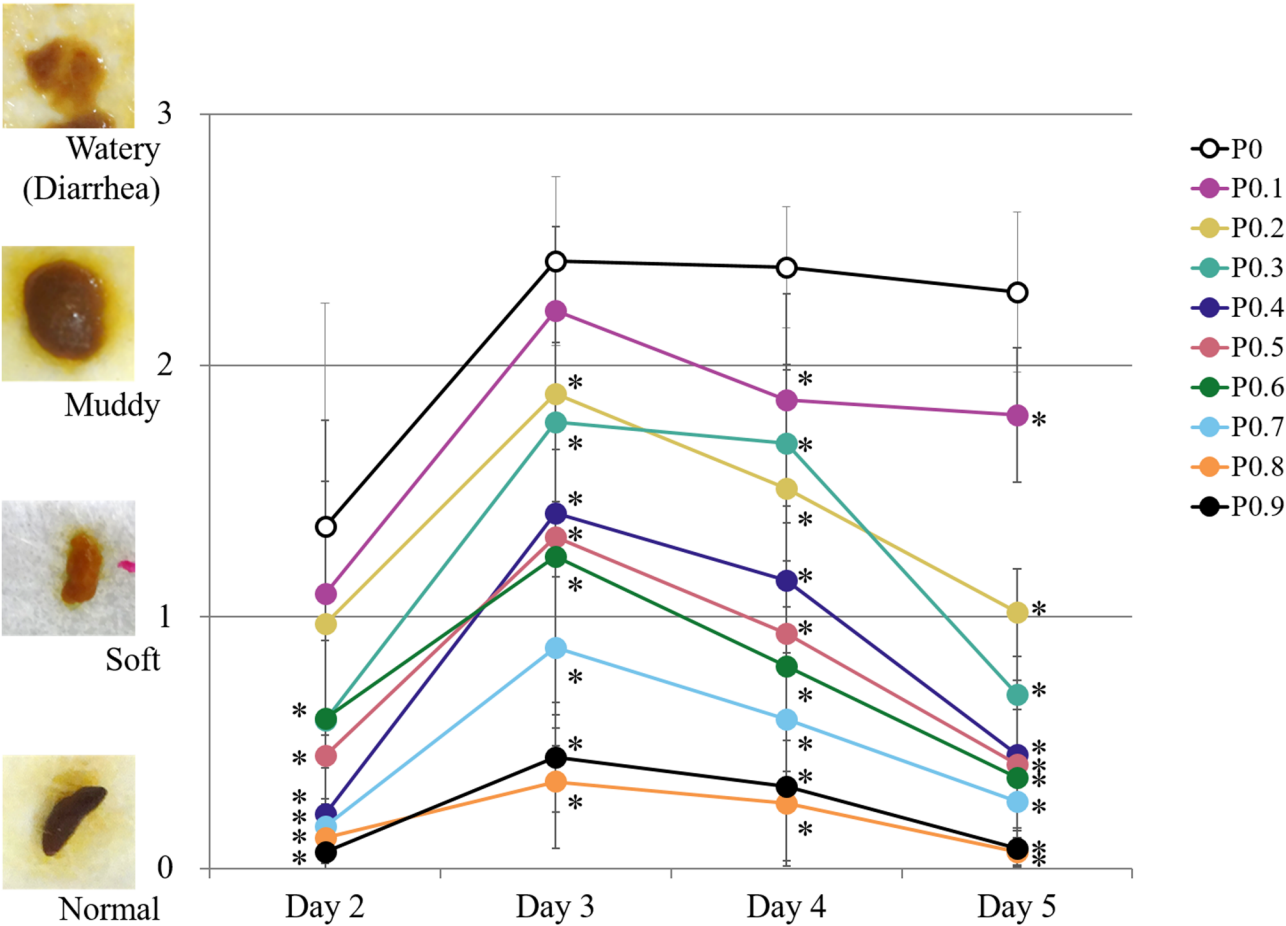
Fecal consistency during the study period. Results are presented as the mean ± SD (*n* = 7–9). Williams’ test or Shirley–Williams’ test: *, *p* < 0.025; n.s., not significant (vs. P0 as control group)

Figure 1 shows the changes in fecal consistency. On Day 4 and thereafter, the fecal scores were significantly lower in all groups fed a pectin-containing liquid diet compared to the P0 group (Day 4: P0, 2.4 ± 0.2; P0.1, 1.9 ± 0.4; P0.2, 1.5 ± 0.5; P0.3, 1.7 ± 0.6; P0.4, 1.1 ± 0.6; P0.5, 0.9 ± 0.6; P0.6, 0.8 ± 0.4; P0.7, 0.6 ± 0.3; P0.8, 0.3 ± 0.3; P0.9, 0.3 ± 0.2. Day 5: P0, 2.3 ± 0.3; P0.1, 1.8 ± 0.3; P0.2, 1.0 ± 0.2; P0.3, 0.7 ± 0.4; P0.4, 0.5 ± 0.3; P0.5, 0.4 ± 0.2; P0.6, 0.4 ± 0.3; P0.7, 0.3 ± 0.2; P0.8, 0.1 ± 0.1; and P0.9, 0.1 ± 0.1; *p* < 0.05). However, as with the P0 group, the P0.1 group largely exhibited muddy or watery feces from Day 3 to Day 5, with a lower frequency of normal feces during this period. Thus, pectin supplementation normalized the fecal consistency of rats given liquid diets via gastric tube feeding, in a dose-dependent manner.

Compared to the P0 group, none of the groups showed a significant difference in body weight on Day 5, but the amount and percentage body weight gain from Day 1 to Day 5 in groups P0.4, P0.5, P0.6, P0.7, P0.8, and P0.9 were significantly greater than in the P0 group (P0, 10.8 ± 4.7; P0.4, 19.7 ± 5.4; P0.5, 16.8 ± 12.6; P0.6, 24.0 ± 11.4; P0.7, 24.0 ± 11.4; P0.8, 24.0 ± 10.7; and P0.9, 23.4 ± 5.9 g; *p* < 0.05, Table 1). Cecum content weight was the same regardless of the amount of pectin.

**Table 1:** Body weight, cecum content weight and blood biochemical tests of rats fed liquid diets containing different amounts of pectin

		P0		P0.1		P0.2		P0.3		P0.4		P0.5		P0.6		P0.7		P0.8		P0.9	
		Mean ± SD		Mean ± SD		Mean ± SD		Mean ± SD		Mean ± SD		Mean ± SD		Mean ± SD		Mean ± an		Mean ± SD		Mean ± SD	
Body weight (Day 1)	g	273.0 ± 10.1		269.8 ± 14.7	n.s.	272.6 ± 14.7	n.s.	271.9 ± 11.1	n.s.	272.9 ± 10.5	n.s.	276.9 ± 11.2	n.s.	274.6 ± 11.6	n.s.	267.4 ± 9.9	n.s.	268.4 ± 11.4	n.s.	269.4 ± 7.5	n.s.
Body weight (Day 5)	g	283.8 ± 11.3		285.3 ± 16.8	n.s.	293.1 ± 12.7	n.s.	288.3 ± 9.7	n.s.	292.6 ± 10.0	n.s.	293.5 ± 8.8	n.s.	291.4 ± 14.5	n.s.	291.4 ± 11.2	*	292.5 ± 8.9	n.s.	292.8 ± 8.2	n.s.
Weight gain	g	10.8 ± 4.7		15.5 ± 4.9	n.s.	20.6 ± 14.5	n.s.	16.4 ± 13.4	n.s.	19.7 ± 5.4	*	16.6 ± 8.8	*	16.8 ± 12.6	*	24.0 ± 11.4	*	24.0 ± 10.7	*	23.4 ± 5.9	*
Weight gain	%	3.9 ± 1.7		5.7 ± 1.8	n.s.	7.7 ± 5.6	n.s.	6.2 ± 5.3	n.s.	7.2 ± 2.1	*	6.1 ± 3.2	*	6.2 ± 4.4	*	9.0 ± 4.4	*	9.1 ± 4.3	*	8.7 ± 2.3	*
Cecum content weight	g	4.4 ± 0.9		5.0 ± 1.4	n.s.	5.0 ± 0.9	n.s.	5.0 ± 1.3	n.s.	4.4 ± 0.6	n.s.	4.2 ± 0.7	n.s.	4.6 ± 1.5	n.s.	4.5 ± 1.0	n.s.	4.1 ± 0.6	n.s.	5.3 ± 1.1	n.s.
TP	g/dL	5.6 ± 0.3		5.4 ± 0.2	n.s.	5.3 ± 0.3	*	5.4 ± 0.3	*	5.4 ± 0.3	*	5.4 ± 0.3	*	5.3 ± 0.2	*	5.3 ± 0.2	*	5.2 ± 0.1	*	5.4 ± 0.2	*
ALB	g/dL	3.3 ± 0.2		3.4 ± 0.2	n.s.	3.3 ± 0.2	n.s.	3.4 ± 0.2	n.s.	3.3 ± 0.3	n.s.	3.5 ± 0.2	n.s.	3.2 ± 0.2	n.s.	3.3 ± 0.2	n.s.	3.3 ± 0.1	n.s.	3.3 ± 0.2	n.s.
BUN	mg/dL	11.9 ± 2.4		11.1 ± 1.0	n.s.	10.9 ± 1.5	n.s.	10.6 ± 1.2	n.s.	10.6 ± 1.6	n.s.	10.7 ± 1.4	n.s.	10.9 ± 1.3	n.s.	10.5 ± 1.0	n.s.	9.8 ± 0.8	*	10.6 ± 0.9	*
CRE	mg/dL	0.2 ± 0.0		0.2 ± 0.0	n.s.	0.3 ± 0.0	n.s.	0.3 ± 0.0	n.s.	0.2 ± 0.0	n.s.	0.2 ± 0.0	n.s.	0.2 ± 0.0	n.s.	0.2 ± 0.0	n.s.	0.2 ± 0.0	n.s.	0.2 ± 0.0	n.s.
Na	mEq/L	137.3 ± 1.2		137.4 ± 0.5	n.s.	137.0 ± 1.2	n.s.	137.8 ± 1.3	n.s.	138.0 ± 1.0	n.s.	138.0 ± 1.1	n.s.	137.9 ± 0.8	n.s.	138.0 ± 0.7	n.s.	138.8 ± 0.7	*	138.3 ± 1.0	*
K	mEq/L	4.9 ± 0.4		4.8 ± 0.6	n.s.	5.0 ± 0.3	n.s.	4.7 ± 0.5	n.s.	4.7 ± 0.4	n.s.	4.9 ± 0.4	n.s.	4.9 ± 0.5	n.s.	4.7 ± 0.4	n.s.	4.7 ± 0.4	n.s.	5.0 ± 0.6	n.s.
Cl	mEq/L	99.7 ± 1.5		101.3 ± 0.9	*	101.7 ± 1.1	*	101.1 ± 0.8	*	101.0 ± 1.6	*	101.4 ± 1.5	*	102.0 ± 1.4	*	101.7 ± 1.6	*	102.9 ± 0.9	*	102.8 ± 1.6	*
IP	mg/dL	7.3 ± 0.4		7.4 ± 0.3	n.s.	7.7 ± 0.2	n.s.	7.4 ± 0.4	n.s.	7.5 ± 0.4	n.s.	7.5 ± 0.2	n.s.	7.5 ± 0.3	n.s.	7.6 ± 0.3	n.s.	7.8 ± 0.3	*	7.7 ± 0.5	*

Results are presented as the mean ± SD (*n* = 7–9). Williams’ test or Shirley–Williams’ test: *, *p* < 0.025; n.s., not significant (vs. P0 as control group). ALB, albumin; BUN, blood urea nitrogen; Cl, Chlorine; CRE, creatinine, IP, inorganic phosphorus; K, Potassium; Na, Sodium; TP, total protein

**Fig. 2:**
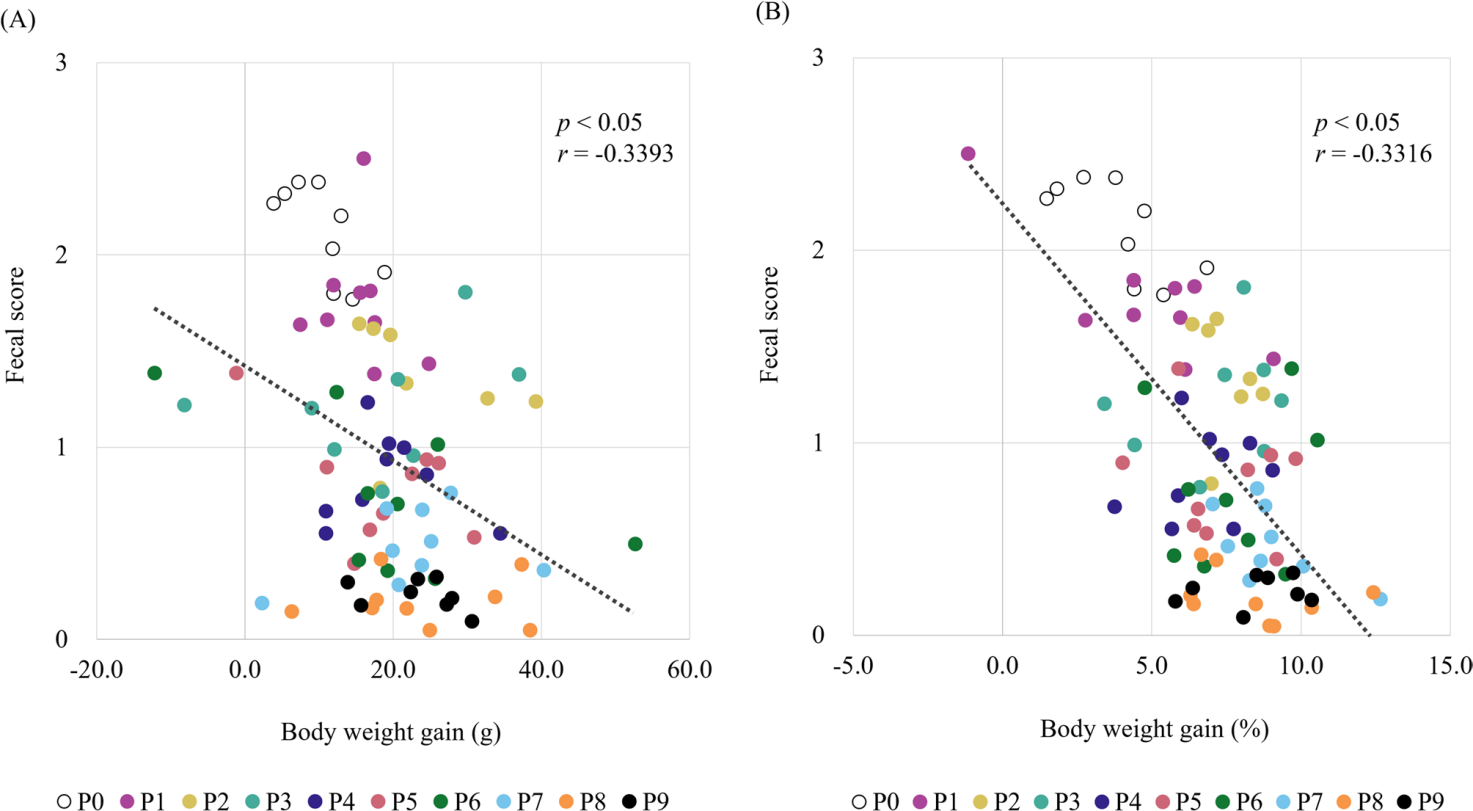
Correlation graph. (**A**) Correlation between fecal consistency and weight gain. (**B**) Correlation between fecal consistency and rate of weight gain. Pearson’s correlation coefficient

Furthermore, this study revealed that the fecal scores of rats fed a liquid diet were significantly correlated with body weight gain (*r* = − 0.3393, *p* < 0.05, Fig. 2). Thus, rats with lower fecal consistency scores than that of the control group showed a greater amount and rate of body weight gain.

Blood biochemical testing did not reveal significant differences in plasma ALB concentrations between the P0 group and any other groups (Table 1). With the exception of the P0.1 group, plasma TP was significantly lower in all groups than the P0 group (P0, 5.6 ± 0.3; P0.2, 5.3 ± 0.3; P0.3, 5.4 ± 0.3; P0.4, 5.4 ± 0.3; P0.5, 5.4 ± 0.3; P0.6, 5.3 ± 0.2; P0.7, 5.3 ± 0.2; P0.8, 5.2 ± 0.1; and P0.9, 5.4 ± 0.2 g/dL; *p* < 0.05, Table 1). BUN was significantly lower in the P0.8 and P0.9 groups than the P0 group (P0, 11.9 ± 2.4; P0.8, 9.8 ± 0.8; and P0.9, 10.6 ± 0.9 mg/dL; *p* < 0.05, Table 1). Significant differences were observed in plasma Na and inorganic phosphorus between the P0.8 and P0.9 groups and the P0 group (Na: P0, 137.3 ± 1.2; P0.8, 138.8 ± 0.7; and P0.9, 138.3 ± 1.0 mEq/L. IP: P0, 7.3 ± 0.4; P0.8, 7.8 ± 0.3; and P0.9, 7.7 ± 0.5 mg/dL; *p* < 0.05, Table 1). Cl was significantly lower in all groups than the P0 group (P0, 99.7 ± 1.5; P0.1, 101.3 ± 0.9; P0.2, 101.7 ± 1.1; P0.3, 101.1 ± 0.8; P0.4, 101.0 ± 1.6; P0.5, 101.4 ± 1.5; P0.6, 102.0 ± 1.4; P0.7, 101.7 ± 1.6; P0.8, 102.9 ± 0.9; and P0.9, 102.8 ± 1.6 g/dL; *p* < 0.05, Table 1).

## Discussion

The present study investigated the effects of fecal consistency on body weight by feeding gastrostomized rats with liquid diets that contained gradually increasing amounts of pectin. Our findings suggest that having lower fecal consistency scores than that of the control group during tube-feeding management contributes to body weight gain during the feeding period.

This suggests that the P0 group, as well as the groups fed a liquid diet with low pectin content, had poor digestion and absorption due to the deterioration of fecal consistency, resulting in difficulty in body weight gain. Thus, fecal consistency may affect body weight in nutritional management by tube feeding. Although many have reported the importance of managing fecal consistency in patients who are tube-fed, few studies have investigated the impact of intervention on body weight. Body weight loss can cause bedsores [10] and loss of skeletal muscle mass [11] in patients receiving enteral nutrition, which may reduce their QoL and affect prognosis. By contributing to the maintenance and gain of body weight, the management of fecal consistency is important for QoL and life prognosis of these patients [12].

In this study, no changes in plasma ALB were observed, likely due to the short test period of five days, which may have prevented any effects from being confirmed. Changes in plasma TP and BUN were noted, and elevated levels of plasma TP and BUN have been reported in dehydration states [13, 14]. This suggests that the high values in the P0 group might indicate a tendency toward dehydration associated with the deterioration of fecal condition. Furthermore, the factors causing changes in electrolytes suggest that in the P0 group, the worsening of fecal condition may have led to a tendency toward dehydration and resultant electrolyte imbalances. This implies that improving fecal condition may contribute to preventing dehydration.

In conclusion, this study suggests that maintaining good fecal condition during the period of enteral feeding management may contribute to weight gain and the prevention of dehydration.

## Limitations

This study has several limitations. First, the intervention duration was five days, which was insufficient to adequately evaluate the effects on body weight gain. Therefore, longer-term studies are needed to better examine the relationship between the maintenance of body weight and fecal consistency. Second, we tested the effect of pectin on fecal consistency but did not examine whether similar results could be obtained with other dietary fibers or antidiarrheal pharmaceutical drugs. Moreover, while we demonstrate that the maintenance of close to normal fecal consistency contributes to weight gain in rats fed a liquid diet via gastric tube-feeding, an extrapolation to humans is difficult. Additional studies are needed to further examine the relationship between maintenance of body weight and fecal consistency in patients receiving nutrition via gastric tube feeding.

## Electronic supplementary material

Below is the link to the electronic supplementary material.

Supplementary Material 1

## Data Availability

The nutritional composition of the liquid diet used in this study is provided in the supplementary information files.

## Abbreviations

ALB: Albumin
BUN: Blood urea nitrogen
Cl: Chlorine
CRE: Creatinine
IP: Inorganic phosphorus
K: Potassium
Na: Sodium
QoL: Quality of life
TP: Total protein
