# Supplementary material for: Effects of managing fecal consistency on body weight in rats given liquid diets with pectin via tube feeding
Source: BMC Res Notes. 2025 Jul 16;18:303. doi: 10.1186/s13104-025-07363-4 (PMC12265129; doi:10.1186/s13104-025-07363-4)
Supplement: Supplementary file 1 — Supplementary Material 1 [file 13104_2025_7363_MOESM1_ESM.docx]

Supplementary Material

Table S1: Nutritional values and ingredients of HINEX E-Gel

|  | Ingredients |  | HINEX E-Gel |
| --- | --- | --- | --- |
| Energy |  | kcal | 100 |
| Volume |  | mL | 125 |
| Protein | Enzyme decomposed soybean protein, Porcine collagen peptide (gelatin), Amino acids | g | 4.0 |
| Fat | Vegetable oils, Medium-chain triglycerides | g | 2.2 |
| Carbohydrates | Maltodextrin, Dietary fiber | g | 16.8 |
| Dietary fiber | Pectin,  Polysaccharide thickener,  Crystalline cellulose | g | 1.4 (Pectin: 0.9) |
| Water |  | g | 110 |
